# Crossing Gender Boundaries: Exploring the Chain-Mediated Causal Role of Social Media Sharing in Shaping Interpersonal Networks and Enhancing Job Satisfaction

**DOI:** 10.3390/bs15010074

**Published:** 2025-01-16

**Authors:** Xin Liu, Nan Qin, Xiaochong Wei

**Affiliations:** School of Public Administration and Policy, Renmin University of China, Beijing 100872, China; lxin@ruc.edu.cn (X.L.); qinnan@ruc.edu.cn (N.Q.)

**Keywords:** gender, social media sharing frequency, interpersonal relationships, job satisfaction, causal path analysis

## Abstract

The theoretical relationship between social media use and job satisfaction, especially concerning gender-specific mechanisms, remains a subject of ongoing debate in the literature. This divergence reflects our insufficient understanding of the complex relationships among gender, social media use, and job satisfaction. Drawing on Social Role Theory (SRT) and the Theory of Planned Behavior (TPB), this study utilizes 4651 valid samples from the 2020 China Family Panel Studies (CFPS) database to investigate how gender influences interpersonal relationships through social media sharing frequency, thereby enhancing job satisfaction. The findings indicate that women, compared to men, exhibit higher job satisfaction and more frequent social media sharing behavior. Moreover, the frequency of social media sharing positively affects job satisfaction by improving interpersonal relationships. This study employs a chain-mediated causal path analysis to delve into the causal relationships among gender, social media sharing frequency, and interpersonal relationships, effectively addressing previous limitations in handling multiple mediating effects. The findings not only provide new insights into the role of social media in the modern workplace but also offer empirical evidence and practical guidance for organizations on leveraging social media to foster employee relationships and enhance job satisfaction.

## 1. Introduction

Public social media platforms in China, including WeChat, TikTok, and Weibo, have fundamentally transformed both personal and professional spheres. These platforms provide diversified spaces similar to “Moments”, where users can record and share their life status and work experiences through various forms including text, images, music, and videos. Notably, mainstream international social media platforms offer similar features, such as Facebook’s “News Feed”, Instagram’s “Feed”, Twitter’s “Timeline”, Snapchat’s “Story”, and WhatsApp’s “Status”. The “Moments” feature not only effectively complements face-to-face communication but also enhances intimacy between users and their friends (Dai et al., 2021). Empirical evidence suggests that user sharing behaviors on these platforms contribute to positive emotional outcomes and foster the development of interpersonal relationships (Manjunatha, 2013), thereby positively impacting both work and life (Choi & Toma, 2014). While social media’s workplace influence continues to grow, scholarly debate persists regarding the mechanisms through which gender mediates the relationship between social media use and job satisfaction. On the one hand, some studies suggest that the proliferation of digital technology is narrowing gender differences (Castolo et al., 2024); on the other hand, other research indicates that traditional gender roles continue to significantly influence social media usage behaviors and their effects (Jiang & Hu, 2016).

A critical review of the existing literature reveals three substantial gaps in our understanding of how social media influences job satisfaction. First, the existing literature predominantly focuses on the direct effects of enterprise social media on job satisfaction and work performance (Lee & Lee, 2022; Robertson & Kee, 2017), with relatively less attention paid to public social media. While enterprise social media directly impacts workplace dynamics, public social media tends to exert its influence through more subtle, indirect pathways (Demircioglu, 2018), yet the underlying mechanisms remain inadequately explored. Second, although previous studies have indicated that gender significantly influences both social media usage (Sun et al., 2018) and job satisfaction (Robertson & Kee, 2017), there is a lack of systematic explanation of the specific pathways through which gender affects job satisfaction via social media use, leading to incomplete understanding of the underlying mechanisms. Third, existing research typically treats gender as a control or moderating variable (Zhang et al., 2019), overlooking its potential role as a key predictor variable in shaping social media usage patterns. This approach limits our deeper understanding of gender’s central role in social media use.

Social Role Theory (SRT) provides a crucial theoretical perspective for understanding how gender influences job satisfaction through social media use. This theory has been widely validated in analyzing how gender differences affect social media use and sharing behaviors (Lin et al., 2013; Chai et al., 2011). SRT posits that men and women assume different roles in society, driven by differing sociocultural expectations, which may influence their social behaviors, attitudes, and emotions (Archer, 1996; Eagly & Wood, 1991). These social role distinctions manifest in differential patterns of social media information sharing. Therefore, SRT provides a theoretical foundation for analyzing how gender influences differences in Moments sharing frequency on social media; additionally, the theory reveals how gender roles determine societal expectations of male and female behavior, specifically how beliefs about appropriate behavior for both genders are shaped by gender roles (Eagly, 1987), which effectively explains gender differences in job satisfaction. As mentioned above, while controversy still exists in applying Social Role Theory (SRT) to explain and validate gender differences in job satisfaction, this paper provides new evidence based on nationwide survey samples from China, while also validating the mediating role of social media usage behavior between the two. Compared to previous research using small-scale survey samples, this study demonstrates significant advantages.

To thoroughly investigate the chain-mediated mechanism of how gender differences in Moments sharing frequency affect job satisfaction through interpersonal relationships, this study employs the Theory of Planned Behavior (TPB) for analysis. TPB was developed by Ajzen (1985, 1991) based on the Theory of Reasoned Action (TRA). Specifically, Ajzen (1985) incorporated perceived behavioral control (PBC) into TRA and renamed it the Theory of Planned Behavior; thus, TRA remains within the scope of TPB. Since TRA concepts will be referenced in subsequent analysis, this explanation is provided. TPB integrates personal attitudes, subjective norms, and perceived behavioral control into a comprehensive framework for understanding human behavior. TPB posits that human behavior is the result of careful deliberation and planning (Ajzen, 1991). The main reason for choosing TPB as the theoretical foundation of this study is its ability to test specific factors in social media and understand their roles in information sharing processes (Fishbein & Ajzen, 2011). Additionally, this theory helps explain behavioral choices in social sharing (Fishbein & Ajzen, 1975), and previous research has demonstrated that incorporating TRA into social media information-sharing behavior studies shows good adaptability (Kim et al., 2016; Raza et al., 2020). Although TPB is considered a comprehensive model for predicting planned individual behavior, empirical studies investigating the impact of social media usage behavior on work outcomes have not fully utilized this theoretical framework. In light of this, based on TPB, this study aims to explore how planned social media use affects interpersonal relationships and subsequently analyze the role of interpersonal relationships in job satisfaction, with the goal of testing and extending TPB’s application in research on how social media usage behavior impacts the workplace domain.

This study pursues three primary objectives: first, to examine the significance of gender-based variations in job satisfaction; second, to explore the mediating role of social media sharing behavior in the relationship between gender and job satisfaction; and third, to examine the chain-mediated effect of interpersonal relationships in this process. The theoretical contribution of this study lies in its pioneering systematic explanation of the mechanism through which gender influences job satisfaction via social media, providing new perspectives for understanding gender roles in the digital era. In terms of practical implications, this study provides empirical evidence for organizations to develop social media usage strategies tailored to employees of different genders. To achieve these objectives, this study will conduct empirical testing using causal path analysis based on national survey data from China Family Panel Studies (CFPS).

## 2. Research Rationale and Research Hypotheses

### 2.1. Research Rationale

The relationship between social media use and workplace satisfaction has emerged as a contentious issue in contemporary research, with particular attention focused on gender-specific mechanisms. These divergent perspectives highlight current gaps in our understanding of the intricate interplay among gender, social media use, and job satisfaction. An extensive examination of the literature demonstrates a clear evolutionary trajectory in scholarly understanding of this phenomenon, characterized by three distinct developmental phases. The first phase was the “gender-blind period”, during which researchers generally believed that the proliferation of digital technology would eliminate traditional gender role boundaries. This perspective was primarily based on technological determinism, which posited that digital transformation would reshape social relationships and diminish gender differences (Jarvenpaa & Staples, 2000). However, this view overlooked the fact that technology use itself might be profoundly influenced by existing social structures, and failed to adequately consider the role of sociocultural factors in technology application.

Subsequently came the “gender awakening period”, during which researchers began to notice significant gender differences in social media usage patterns. However, research during this period had two typical limitations: first, gender was often simply treated as a control or moderating variable, overlooking its potential predictive role; second, explanations of gender differences remained superficial, lacking investigation into deeper mechanisms (Krasnova et al., 2017; Van Zoonen et al., 2017). This approach reflected researchers’ insufficient understanding of gender roles’ continuing influence in the digital era and failed to deeply analyze gender’s core role in social media use.

More recently, the field has entered a “gender integration period”, where research has begun to focus on gender’s active role in social media use. This shift stems from three significant findings: first, digital technology, rather than eliminating gender differences, has provided new spaces for gender role expression; second, gender differences demonstrate unique patterns across different cultural contexts; and third, social media use may reinforce rather than weaken traditional gender role characteristics (Zhang et al., 2019). This evolution in understanding reveals three deep-seated theoretical challenges. The first is the “persistence puzzle”, questioning whether the gender differences emphasized by Social Role Theory maintain their explanatory power in social media contexts, which involves fundamental understanding of the interaction between technology and social relationships. The second is the “mechanism puzzle”, examining how, if gender differences indeed exist, they influence job satisfaction through psychological and social mechanisms, particularly questioning whether traditional explanatory frameworks remain applicable in new scenarios where virtual and physical realities intersect. The third is the “universality puzzle”, investigating whether these influence mechanisms demonstrate consistency across different cultural contexts or exhibit significant cultural specificity.

More specifically, existing research exhibits four key deficiencies. First, there is insufficient systematic theoretical integration; although previous studies have separately applied Social Role Theory and the Theory of Planned Behavior, they lack deep consideration of the organic combination of these two theoretical frameworks, particularly regarding their applicability in digital contexts. Second, there is a lack of causal chain completeness, namely insufficient theoretical explanation and empirical testing of how gender differences influence job satisfaction through specific pathways. Third, there is inadequate cultural context embeddedness, specifically a lack of in-depth exploration of unique gender role manifestations in East Asian cultural contexts. Finally, there is insufficient methodological precision, as traditional analytical methods struggle to effectively address multiple mediation and endogeneity issues.

To overcome these limitations, this study attempts to make the following theoretical innovations. First, it constructs a “dual-core theoretical framework”, using Social Role Theory as the foundational framework for explaining gender differences while incorporating the Theory of Planned Behavior to explain specific behavioral mechanisms, achieving organic theoretical integration. Second, it proposes a “chain-mediated mechanism”, systematically revealing the pathways through which gender influences job satisfaction via social media sharing frequency and interpersonal relationships. Finally, it adopts an innovative causal path analysis method to enhance the reliability of the research findings. Furthermore, given the commonalities and differences between China and the West in social media use, this study’s conclusions not only draw upon Western research findings but also incorporate China’s unique cultural context, aiming to provide a more comprehensive and universally applicable theoretical understanding that offers valuable reference for research across different cultural contexts.

### 2.2. Research Hypotheses

#### 2.2.1. Gender and Job Satisfaction

Social Role Theory (SRT) provides an important theoretical foundation for understanding the role of gender in job satisfaction. The theory posits that men and women play different roles in work and family environments, with these differences stemming from societal and cultural expectations, which in turn shape their behaviors and attitudes (Eagly, 1987). While traditional research has often treated gender as a control or moderating variable, Social Role Theory emphasizes that gender role differentiation is achieved through power and resource allocation mechanisms within social structures, with socialization processes further reinforcing expectations of gender-specific behaviors (Eagly & Wood, 1991, 2016). In organizational contexts, these gender role expectations further interweave with work roles, creating unique gender–work interaction patterns.

Research has found that men tend to pursue external rewards (such as income and status), while women place greater emphasis on emotional support and work–life balance. This difference in tendencies can be understood at two levels: at the macro level, it reflects institutionalized societal expectations for different genders; at the micro level, it embodies internalized value orientations developed through the socialization process. Consequently, women find it easier to obtain emotional compensation through family roles (Q. Huang & Gamble, 2015). The mechanism of job satisfaction formation shows significant gender differences, primarily manifested in three aspects. First, women often view work as one of multiple pathways to achieve self-worth, rather than the only path (Westover & Andrade, 2024); second, women are more adept at acquiring emotional resources and social support through multiple roles (Flaherty & Richman, 1989); finally, women’s evaluation criteria for work are more diverse, encompassing not only objective material rewards but also subjective emotional experiences and interpersonal interactions (Ross & Mirowsky, 1996). Even when at a relative disadvantage in terms of compensation and promotion opportunities, women often report higher job satisfaction than men (Hagedorn, 1995).

In the digital era, the proliferation of social media has provided a new platform for gender role expression. Social Role Theory has been further developed in the social media environment: first, social media has reduced social constraints on emotional expression, making it easier for gender roles to be reinforced in virtual spaces (Reychav et al., 2019; Kivran-Swaine et al., 2012); second, the interactive characteristics of social media highly align with women’s socialization characteristics, providing them with more suitable channels for obtaining emotional support (Li & Zhuo, 2023). Research indicates that women are more inclined to engage in positive interactions on social media, aiming to establish and maintain extensive interpersonal networks (Dai et al., 2021; Laor, 2022). By frequently sharing positive aspects of their lives and work, women can gain more emotional support and trust (Nathan et al., 1991), which in turn positively influences their job satisfaction (L. V. Huang & Liu, 2017). In contrast, men’s use of social media tends to focus more on information acquisition and task-oriented activities, with weaker connections to interpersonal interactions.

Based on the above analysis, this study posits that gender role differences ultimately lead to variations in job satisfaction through their influence on individuals’ social media usage patterns and their ability to obtain emotional support. This influence not only reflects the continuation of traditional gender roles but also demonstrates new characteristics of gender role expression in the digital era. Therefore, the following hypothesis is proposed:

**Hypothesis** **1:** *In the context of social media, women exhibit significantly higher job satisfaction than men.*

#### 2.2.2. Social Media Sharing Behavior and the Chain-Mediated Role in Interpersonal Networks

Social Role Theory (SRT) not only explains the formation mechanism of gender differences but, more importantly, reveals the stability and universality of gender as a predictor variable. This predictive effect is particularly evident in social media sharing behavior, as social media provides individuals with a relatively free platform for self-expression, allowing internalized gender role characteristics to manifest more naturally (Suman et al., 2022). Men and women assume different roles in society, and this role differentiation leads to significant differences in behavioral patterns (Eagly, 1987; Eagly & Wood, 1991; Eagly et al., 2000). Traditionally, women tend to take on more family caregiving responsibilities, while men are typically viewed as the primary economic providers.

This deeply ingrained role division not only influences offline behavior but also extends into cyberspace. Social Role Theory’s concept of ‘role spillover effect’ demonstrates how behavioral patterns established in one domain systematically manifest in other contexts, an effect that is particularly evident in the digital era (Piszczek et al., 2016). Specifically, women’s tendencies toward emotional communication and social interaction, formed in traditional social roles, naturally transform into more frequent sharing and interactive behaviors on social media (Li & Zhuo, 2023; Reychav et al., 2019; Cirucci, 2018). This explains why women place greater emphasis on emotional communication and social interaction and are more inclined to share life experiences on social media to maintain and expand their interpersonal networks (Eagly & Wood, 2016). Research indicates that women exhibit significantly higher frequencies of interactive behaviors on social media, such as sharing, liking, and commenting, compared to men (Dai et al., 2021; Laor, 2022).

The gender differences in social media sharing behavior can be deeply understood through two complementary theoretical frameworks: the Theory of Reasoned Action (TRA) and the Theory of Planned Behavior (TPB). The Theory of Reasoned Action suggests that individual behaviors are often based on anticipated outcomes and others’ expectations (Fishbein & Ajzen, 2011). This theory emphasizes the social constructiveness of behavioral motivation, namely that individual behavioral choices are significantly constrained by social norms and role expectations. In the social media environment, this social constructiveness has been newly reinforced through digital interactions (Karnowski et al., 2018). When users share content on social media, they hope to strengthen emotional connections through social feedback such as likes and comments (Yawen et al., 2017). For instance, research has found that the primary motivation for social media users to share is maintaining interpersonal interactions and gaining recognition from others (Lee et al., 2016). The Theory of Planned Behavior further explains how social media sharing influences job satisfaction through improved interpersonal relationships. This theory emphasizes that behavior is driven by three factors: attitudes, subjective norms, and perceived behavioral control. Regarding attitudes, women demonstrate more positive tendencies toward social media sharing, stemming from their relationship-oriented characteristics formed during socialization. This attitude is reflected not only in the frequency of sharing behavior but also in the quality and emotional depth of shared content (Lin & Wang, 2020). Research indicates that women tend to share positive events, aiming to enhance their self-image and strengthen social connections through gaining more likes and comments (Qiu et al., 2012). This positive sharing not only boosts self-esteem but also helps gain more support and trust in the workplace (Nathan et al., 1991).

Regarding subjective norms, behavioral standards are distinctly shaped by gender role expectations. Women are more susceptible to perceived expectation pressure from social networks, which in turn reinforces their sharing behavior (Eagly & Crowley, 1986). Subjective norms refer to individuals’ perceptions of expectations from important others (Ajzen, 1991). In the workplace, positive social interactions are viewed as crucial for team integration and enhanced collaboration (Pless & Maak, 2004). When women’s social media sharing behaviors receive positive responses from colleagues and supervisors, this recognition promotes stronger relationships with colleagues (Rode, 2016; Wang & Lei, 2023). Regarding perceived behavioral control, gender socialization profoundly influences individuals’ perceptions of their own capabilities. Perceived behavioral control refers to individuals’ perceptions of their abilities, resources, and opportunities to perform specific behaviors (Ajzen, 1991). Women’s stronger sense of self-efficacy in social media environments largely stems from their social skills and emotional expression abilities cultivated during socialization (Li & Zhuo, 2023). This self-efficacy makes them more likely to use social media to gain social support (Ridgway & Clayton, 2016; Gu et al., 2021). Studies also indicate that by sharing positive events, women can enhance others’ impressions of them, thereby gaining stronger social support, which further boosts their job satisfaction (Guinot et al., 2014; Thoms et al., 2002).

In summary, Social Role Theory, the Theory of Reasoned Action, and the Theory of Planned Behavior collectively construct a comprehensive theoretical framework that explains why gender should be viewed as a predictor variable rather than a simple control or moderating variable. These three theories reveal, from different perspectives, how gender systematically influences individuals’ social media sharing behavior and its effects through deep socialization mechanisms. Therefore, this study proposes the following hypothesis:

**Hypothesis** **2:** *In the context of social media, women exhibit significantly a higher frequency of social media sharing than men, and this sharing frequency can enhance job satisfaction through the promotion of interpersonal relationships.*

**Figure 1:**
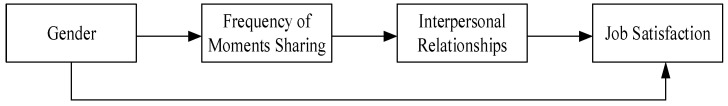
Theoretical model diagram.

Figure 1 illustrates the theoretical model of this study.

## 3. Research Methodology

### 3.1. Sample and Data

All data used in this study are sourced from the China Family Panel Studies (CFPS) database. CFPS is a large-scale longitudinal survey that began in 2010, releasing survey data every two years. Since data related to “Moments” sharing behavior was only collected starting in 2020, this study is based on the 2020 data (as the 2022 survey data have not yet been released). To ensure data quality and analytical robustness, we implemented comprehensive data cleaning and filtering procedures, resulting in 4651 valid data entries.

### 3.2. Variable Measurement

Job Satisfaction: The original survey question was “Overall, how satisfied are you with this job?” (For discussions on the use of single-item measures in research, see Demerouti et al., 2012; Trougakos et al., 2008.) Responses were rated on a scale where 1 represents “Very dissatisfied”, 2 represents “Dissatisfied”, 3 represents “Neutral”, 4 represents “Satisfied”, and 5 represents “Very satisfied”.

Gender: In the original survey, gender was coded as 0 for female and 1 for male.

Frequency of Moments Sharing: The original survey question was “How frequently do you share your work or life on Moments?” Responses were coded as follows: 1 for “Almost every day”, 2 for “3–4 times a week”, 3 for “1–2 times a week”, 4 for “2–3 times a month”, 5 for “Once a month”, 6 for “Once every few months”, and 7 for “Never”. The variable was reverse-coded for analysis.

Quality of Social Relationships (Score): The original survey question was “How would you rate the quality of your social relationships?” This was a rating scale item, with scores ranging from 0 (lowest) to 10 (highest).

Control Variables: Drawing on the findings and standards of previous studies, this study controls for variables such as education level, age, marital status, social status, local income conditions (Laor, 2022), presence of direct subordinates (Zhang et al., 2019), income and type of employer (Liang et al., 2021), health status, weekly working hours, one-way commute time, frequency of night shifts, frequency of weekend work, flexibility of working hours (Costa et al., 2006), and household registration status (Fossum, 1974).

The data for this study were obtained from the China Family Panel Studies (CFPS) database. The research primarily focuses on WeChat, China’s most widely used social media platform, which serves a unique dual function as both a public social media platform and an important tool for internal corporate communication. Table 1 details the numerical values and corresponding meanings for each variable.

### 3.3. Causal Path Analysis

The current analysis of mediation mechanisms primarily relies on traditional stepwise testing methods. However, this approach can lead to endogeneity issues in the direct path due to the omission of the mediator variable. Most of the current corrective measures only consider the effect of the treatment variable on the mediator variable, while the effect of the mediator variable on the outcome variable relies on theoretical explanations. Although this approach can avoid endogeneity bias caused by the omission of the mediator variable, it is not comprehensive for the entire mediation analysis. Zhou and Yamamoto (2023) address this limitation through causal path analysis (CPA), a method based on the Rubin causal framework that moves beyond traditional linear regression estimation (Zhou, 2022; Zhou & Yamamoto, 2023). In current causal inference, the Rubin Potential Outcome Model is widely adopted as an analytical framework (Angrist & Pischke, 2009). Under this framework, the potential outcome of individual i is considered as a function of the treatment variable $X_{i}$, denoted as $Y\left(X_{i}\right)$. This framework posits that if the potential outcomes of an individual i both before and after treatment can be observed, then the average treatment effect (ATE) is the average change in potential outcomes for all individuals before and after treatment, mathematically expressed as

(1) ATE=E[Y(1)−Y(0)]

where Y(1) represents the potential outcome when the individual receives treatment, and Y(0) represents the potential outcome when the individual does not receive treatment. In this study, $M_{1}$ and $M_{2}$ are two possible mediator variables in the transmission mechanism, having a logical sequential order and depending on the treatment variable $X_{i}$. Suppose the potential outcome of individual i is a function of $X_{i},M_{1}\left(X_{i}\right)$, and $M_{2}\left(X_{i},M_{1}\left(X_{i}\right)\right)$, i.e.,

(2) $$Y_{i}=Y\left(X_{i},M_{1}\left(X_{i}\right),M_{2}\left(X_{i},M_{1}\left(X_{i}\right)\right)\right)$$

Based on the Rubin causal identification framework, $M_{1}\left(X_{i}\right)$ is regarded as the potential outcome of mediator variable $M_{1}$ under the treatment state $X_{i}$, and $M_{2}\left(X_{i},M_{1}\left(X_{i}\right)\right)$ is the potential outcome of mediator variable $M_{2}$ under the treatment state $X_{i}$ and the mediator variable $M_{1}=M_{1}\left(X_{i}\right)$. Therefore, the treatment variable $X_{i}$ directly affects the potential outcome $Y_{i}$, and it also indirectly affects the potential outcome $Y_{i}$ through the mediator variables $M_{1}$ and $M_{2}$. Based on these assumptions, the average treatment effect can be defined as

(3) $$ATE=E\left[Y\left(1,M_{1}(1),M_{2}\left(1,M_{1}(1)\right)\right)-Y\left(0,M_{1}(0),M_{2}\left(0,M_{1}(0)\right)\right)\right]$$

The above equation essentially adds mediator variables to the basic form of $Y\left(X_{i}\right)$ in Equation (1). According to Zhou (2022) and Zhou and Yamamoto (2023), the average treatment effect equation can be decomposed as follows:

(4) $$\begin{array}{rr} E[Y(1)-Y(0)]= & E\left[Y\left(1,M_{1}(1),M_{2}\left(1,M_{1}(1)\right)\right)-Y\left(0,M_{1}(0),M_{2}\left(0,M_{1}(0)\right)\right)\right]\\ = & \underset{A\to Y}{\underbrace{E\left[Y\left(1,M_{1}(0),M_{2}\left(0,M_{1}(0)\right)\right)-Y\left(0,M_{1}(0),M_{2}\left(0,M_{1}(0)\right)\right)\right]}}\\ & +\underset{A\to M_{2}\to Y}{\underbrace{E\left[Y\left(1,M_{1}(0),M_{2}\left(1,M_{1}(0)\right)\right)-Y\left(1,M_{1}(0),M_{2}\left(0,M_{1}(0)\right)\right)\right]}}\\ & +\underset{A\to M_{1}\to Y;A\to M_{1}\to M_{2}\to Y}{\underbrace{E\left[Y\left(1,M_{1}(1),M_{2}\left(1,M_{1}(1)\right)\right)-Y\left(1,M_{1}(0),M_{2}\left(1,M_{1}(0)\right)\right)\right]}}\\ \equiv & \tau_{A\to Y}+\tau_{A\to M_{2}\to Y}+\tau_{A\to M_{1}⇝Y} \end{array}$$

**Figure 2:**
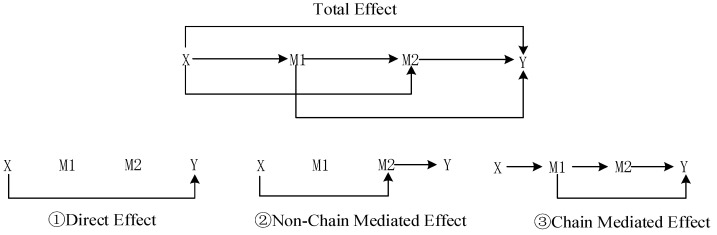
Chain causal path diagram.

In the equation, $E\left[Y\left(1,M_{1}(0),M_{2}\left(0,M_{1}(0)\right)\right)-Y\left(0,M_{1}(0),M_{2}\left(0,M_{1}(0)\right)\right]\right.$ represents the direct effect of X→Y. $E\left[Y\left(1,M_{1}(0),M_{2}\left(1,M_{1}(0)\right)\right)-Y\left(1,M_{1}(0),M_{2}\left(0,M_{1}(0)\right)\right)\right]$ is the non-chain-mediated effect of $X\to M_{2}\to Y$, which means the indirect effect of the treatment variable X on the outcome variable Y via mediator $M_{2}$.$\;E\left[Y\left(1,M_{1}(1),M_{2}\left(1,M_{1}(1)\right)\right)-Y\left(1,M_{1}(0),M_{2}\left(1,M_{1}(0)\right)\right)\right]\;$represents the chain-mediated effect of $X\to M_{1}⇝Y$ (including the paths $X\to M_{1}\to Y$ and $X\to M_{1}\to$ $\left.M_{2}\to Y\right)$. Figure 2 illustrates the theoretically possible causal paths from the treatment variable X to the outcome variable Y: (1) X→Y; (2) $X\to M_{2}\to Y$; (3) $X\to M_{1}⇝Y$.

Following Pearl’s (2009) methodology, a Directed Acyclic Graph (DAG) can be used to represent a non-parametric structural equation model with mutually independent error terms. This model assumes no confounding in the relationships between treatment and mediator (A→M), treatment and outcome (A→Y), and mediator and outcome (M→ Y) after controlling for prior variables. This assumption is much stronger than the standard no-confounding assumption often invoked by researchers in observational studies to identify the average total effect (ATE) (Zhou & Yamamoto, 2023). The key to this assumption is that it includes multiple conditional independence relations, particularly involving the so-called “cross-world counterfactuals”, for example, for any values of $a,a_{1},m_{1},m_{2},Y\left(a,m_{1},m_{2}\right)⊥M_{1}\left(a_{1}\right)|X,A$. If factors that arise after treatment and can influence both the mediator and the outcome are not properly accounted for, the previously assumed clear causal relationships become blurred. Therefore, to reduce bias from potential post-treatment confounding factors, Zhou and Yamamoto (2023) recommend including all observed post-treatment variables in either $M_{1}$ or $M_{2}$, depending on the assumed causal order among the variables.

Under this assumption, the path-specific effects defined by Equation (4) are identifiable in a non-parametric sense. To identify the components included in Equation (1), it is sufficient to identify the counterfactual expectation $E\left[Y\left(a,M_{1}\left(a_{1}\right),M_{2}\left(a_{2},M_{1}\left(a_{1}\right)\right)\right)\right]$ for any combination of $a,a_{1},a_{2}\in \{0,1\}$ (i.e., the expected value of the outcome variable Y given specific values of the mediator variables $M_{1}$ and $M_{2}$ determined by $a_{1}$ and $a_{2}$ under a specific treatment state A). This expectation can be expressed as a function of the observed variables:

(5) $$\begin{array}{ll} & E\left[Y\left(a,M_{1}\left(a_{1}\right),M_{2}\left(a_{2},M_{1}\left(a_{1}\right)\right)\right)\right]\\ = & ∭E\left[Y|x,a,m_{1},m_{2}\right]f\left(m_{2}|x,a_{2},m_{1}\right)f\left(m_{1}|x,a_{1}\right)f(x)dm_{2}dm_{1}dx, \end{array}$$

where f(⋅) represents the probability density/mass function. This formula generalizes Pearl’s (2001) single-mediator formula to the situation of two (or more) causally dependent mediators. It is important to note that $X\to M_{1}\to Y$ and $X\to M_{1}\to M_{2}\to Y$ are difficult to identify individually because, under the assumptions of causal path analysis, $E\left[Y\left(x,M_{1}\left(x_{1}\right),M_{2}\left(x_{2},M_{1}\left(x_{12}\right)\right)\right)\right]$ can only be identified when $x_{12}=x_{1}$ (Zhou, 2022; Zhou & Yamamoto, 2023). The average treatment effect for $X\to M_{1}\to Y$ is expressed as $\tau_{X\to M_{1}\to Y}\left(x,x_{1},x_{12}\right)=$ $E\left[Y\left(x,M_{1}(1),M_{2}\left(x_{2},M_{1}\left(x_{12}\right)\right)\right)-Y\left(x,M_{1}(0),M_{2}\left(x_{2},M_{1}\left(x_{12}\right)\right)\right)\right]$, where the relationship between $x_{12}$ and 0.1 is indeterminate, thus making it unidentifiable. Similarly, the average causal treatment effect for $X\to M_{1}\to M_{2}\to Y$ is $\tau_{X\to M_{1}\to M_{2}\to Y}\left(x,x_{1},x_{2}\right)=E\left[Y\left(x,M_{1}\left(x_{1}\right),M_{2}\left(x_{2},M_{1}(1)\right)\right)-Y\left(x,M_{1}\left(x_{1}\right),M_{2}\left(x_{2},M_{1}(0)\right)\right)\right]$, where the relationship between $x_{1}$ and 0,1 is also indeterminate. However, this can identify the average treatment effect for $X\to M_{1}⇝Y$ because the states of the treatment variables in the equation correspond one-to-one.

A notable limitation of this method is that it requires significant computational power when there are multiple mediators. When there are M ordered mediators (M>1), the direct path from the treatment variable to the outcome variable can combine K mediators in any way, resulting in $2^{M}$ possible paths, each of which can be turned on or off. In the present study, computational analysis using the BART paths() function required approximately 75 h of processing time. If conditions permit, using large-scale computing resources would speed up the process significantly.

Lastly, it is worth noting that the causal path analysis method proposed by Zhou and Yamamoto (2023) can be applied to both observational and experimental data. The mediator variables can be either continuous or categorical. When employing Pure Imputation Estimators, binary treatment variables are not requisite.

Additionally, this method is not limited by the number of mediators. This is particularly important in many fields of research as it expands the applicability of analytical methods, allowing researchers to explore and analyze mediation effects and path-specific effects even in the absence of experimental designs. By providing a clear set of assumptions and statistical models, the authors make it possible to apply these methods to observational data. Through the introduction and application of new statistical methods and models, such as Generalized Linear Models (GLMs), Gradient Boosting Machines (GBMs), Bayesian Additive Regression Trees (BART), and sensitivity analysis techniques like bias adjustment, they demonstrate that even in observational studies, it is possible to effectively identify and estimate causal path effects through multiple mediators.

## 4. Results

### 4.1. Descriptive Statistical Analysis

Table 2 presents the descriptive statistics of the variables, including job satisfaction, gender, frequency of Moments sharing, interpersonal relationships, age, and other key variables. The table reports the sample size, mean, standard deviation, minimum, and maximum values for each variable.

### 4.2. Baseline Regression Analysis

Table 3 presents the baseline regression results of the study. The regression results in the first column indicate that the gender variable has a significant negative impact on the frequency of Moments sharing (β = −0.7249, *p* < 0.001). The second column shows a significant positive correlation between the frequency of Moments sharing and the quality of social relationships (β = 0.0451, *p* < 0.01). Additionally, the second column also shows that the direct effect of gender on social relationships is not significant (β = 0.0317, *p* > 0.05). In the third column, gender has a direct negative impact on job satisfaction (β = −0.0843, *p* < 0.001). Furthermore, the positive effects of the frequency of Moments sharing (β = 0.0252, *p* < 0.001) and interpersonal relationships (β = 0.0356, *p* < 0.001) on job satisfaction are also demonstrated.

Comparative analysis of these three columns reveals that gender, as a key variable, not only directly influences individuals’ frequency of Moments sharing and job satisfaction but also indirectly affects job satisfaction through its impact on the frequency of Moments sharing, which in turn influences interpersonal relationships.

### 4.3. Chain-Mediated Causal Path Analysis

Table 4 presents the results of the chain-mediated causal path analysis. For the analysis, the study employed the Pure Imputation Estimator and the Imputation-Based Weighting Estimator. The results indicate that the findings from both estimators are similar. The total effect results show that, compared to women, men have lower job satisfaction—a decrease of 0.103 on the probability scale. Using the Pure Imputation Estimator, the direct effect is −0.091, suggesting that most of the total effect is attributable to differences between genders. Most of the indirect effects are transmitted through the X-M1-M2-Y path (−0.017), rather than directly through interpersonal relationships (X-M2-Y, 0.004). This finding suggests that gender differences in the frequency of Moments sharing contribute to improved job satisfaction through their influence on interpersonal relationships.

### 4.4. Sensitivity Analysis

In observational studies, treatment (i.e., intervention or exposure) is typically not randomly assigned, which may lead to bias in estimating path-specific effects (PSEs). Even in randomized experimental studies, there may be confounding of the mediator–outcome relationship if unobserved variables simultaneously affect both the mediator and the outcome, potentially compromising the reliability of research findings. To address this issue, this study conducts sensitivity analysis using the bias factor method developed by Zhou and Yamamoto (2023). This method extends VanderWeele’s (2010) bias formula to scenarios with multiple causally dependent mediators. The bias factor method helps researchers understand whether omitting certain unobserved or unmeasured variables that affect both the mediator (such as sharing frequency on social media) and the outcome variable (such as job satisfaction) would bias the estimation of causal relationships. In essence, this method can detect whether ignoring certain variables would make the research findings imprecise. By evaluating such potential bias, researchers can enhance the reliability of their results even when they cannot directly measure all possible influencing factors, while also identifying and adjusting for unobserved confounders that might significantly impact the research findings.

As mentioned earlier, due to excessive missing values, the number of years of work experience was not included in the analysis, and some more important confounding variables, such as personality traits, are not available in the CFPS database (Mouakket & Sun, 2020; Kim & Chung, 2014). To assess the impact of these factors on the robustness of research conclusions, this study conducts sensitivity analysis using the bias factor method developed by Zhou and Yamamoto (2023). The PSE for Moments sharing frequency (τA→M1⇝Y) is evaluated. Assuming the existence of a strong unobserved confounder U (e.g., personality traits) that simultaneously influences the core variables in the model, the estimated effect (PSE) through the Moments sharing frequency (M1) path may be subject to bias when accounting for the influence of the unobserved variable U.

**Figure 3:**
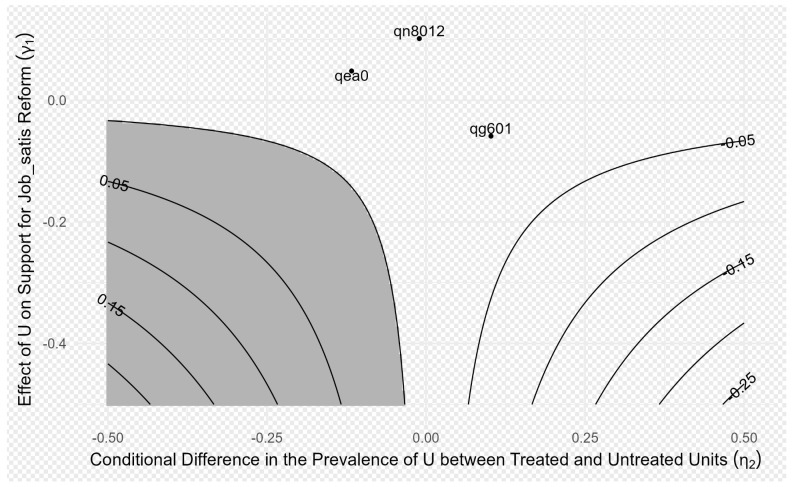
Estimated path-specific effect of gender on job satisfaction through Moments sharing frequency, adjusted for bias.

In Figure 3, different values of γ1 and η1 form contour lines representing the bias-adjusted PSE. The figure also shows the possible values of γ1 and η1 when U has the same effect as three observed covariates (e.g., marital status, social status, and frequency of night shifts). It is evident that only when both γ1 and η1 are relatively large (e.g., when both γ1 and η1 are −0.25) could the original estimated value (−0.017) be entirely attributed to this unobserved confounding. In other words, unless γ1 and η1 both reach considerably large values, the unobserved variable U is unlikely to fully explain the originally estimated effect, indicating that the original research findings are robust to potential unobserved confounding. Additionally, judgment can be made based on whether the sensitivity analysis points fall within the gray area. If the annotation points in the sensitivity analysis (representing the hypothesized influence of specific unobserved variable U) do not fall within the gray area, this suggests that even if such unobserved confounding factors exist, they are unlikely to alter the direction of the research conclusions, thus indicating that the original research findings are relatively robust. In conclusion, the results suggest that these estimates would only be significantly affected under extreme circumstances (i.e., when the influence of the unobserved variable U is very large and significantly differs between treatment and non-treatment groups). This provides support for the credibility of the research findings.

## 5. Discussion

This study explored gender differences in the frequency of Moments sharing and their role in promoting interpersonal relationships, thereby enhancing job satisfaction. The results show that gender, Moments sharing frequency, interpersonal relationships, and job satisfaction form a chain mediation model. The findings indicate that compared to males, females share more frequently on Moments, which positively affects job satisfaction through interpersonal relationships. The direct path also shows that females have higher job satisfaction, forming a mutually reinforcing relationship between the direct and indirect paths. In summary, this study suggests that females may experience higher job satisfaction due to their unique social roles and workplace experiences, such as stronger interpersonal relationships and social support networks. This provides new insights into understanding how gender influences job satisfaction through different mechanisms of social media use.

Compared to existing research, this study demonstrates significant innovations in both methodology and context. First, the data are sourced from the 2020 China Family Panel Studies (CFPS), encompassing 4651 valid samples nationwide, which significantly surpasses the smaller sample sizes common in previous research (e.g., Demircioglu, 2018, with a sample size of 892), thereby enhancing the reliability of statistical inference and the generalizability of results. Second, this study employs a chain-mediated causal path analysis method, effectively addressing the multiple mediation effects and potential endogeneity issues faced in traditional mediation effect analyses. Unlike mediation variable analyses that focus on single paths, chain-mediated analysis offers greater precision and explanatory power in parsing causal pathways. Furthermore, this study focuses on China’s unique social media platform “Moments”, considering both the commonalities with Western platforms (such as Facebook and Twitter) and its unique cultural characteristics when examining the relationship between social media use and workplace performance. This cross-cultural approach enhances methodological rigor while expanding the generalizability of findings across diverse cultural contexts, contributing to a more comprehensive understanding of social media’s influence across different cultural contexts.

### 5.1. Theoretical Contributions

First, this study further validates the universality and effectiveness of Social Role Theory in explaining gender differences through empirical testing within the specific cultural context of China. As a typical representative of the East Asian cultural sphere, China’s cultural characteristics are similar to those of other East Asian countries to some extent. Therefore, the findings of this study may also be applicable to other East Asian countries with similar cultural backgrounds. The empirical analysis reveals that females have higher job satisfaction than males, supporting Eagly’s (2013) view that gender roles and cultural expectations significantly impact job satisfaction. These findings present a significant counterpoint to prior research (Robst et al., 2003; Ehrenberg, 2003; Zoghi, 2003) that found no substantial gender differences in job satisfaction, providing new evidence for the discussion on gender differences in job satisfaction.

Second, this study reveals gender differences in Moments sharing frequency, providing new empirical evidence for the application of Social Role Theory in the digital society context. Social Role Theory (SRT) provides a theoretical framework for analyzing how gender differences influence social media use and sharing behavior. The study found that females significantly outperformed males in Moments sharing frequency, offering a gender-sensitive perspective on understanding how gender shapes individual behavior patterns on social media platforms. Specifically, this finding challenges the gender-neutral assumptions in previous studies on social media usage behavior (Jarvenpaa & Staples, 2000; Acquisti & Gross, 2006), emphasizing the importance of considering gender roles when analyzing social media behavior.

Third, this study extends the application of the Theory of Planned Behavior (TPB), particularly in exploring how social media sharing behavior affects interpersonal relationships and job satisfaction. TPB is typically used to explain the motivation and decision-making process of individual behavior (Ajzen, 1991). This study empirically demonstrates a positive relationship between social media sharing behavior and the quality of interpersonal relationships. This finding not only validates the positive role of social media in promoting interpersonal relationships (Manjunatha, 2013) but also provides theoretical support for understanding how social media connects people in modern society. By applying TPB to social media usage behavior, this study reveals the roles of attitudes, subjective norms, and perceived behavioral control in the decision-making process of social media sharing, providing a theoretical framework for subsequent research to explore the behavioral motivations of social media use and its work-related outcomes.

Finally, this study further investigates interpersonal relationships as a chain mediation mechanism between social media usage behavior and job satisfaction. The results partially validate Demircioglu’s (2018) conclusion that public social media use significantly affects job satisfaction indirectly. The identification of this mediating mechanism advances our theoretical understanding of how social media usage behavior influences the workplace and provides a new application example for TPB in explaining the relationship between complex social behavior and psychological states. Additionally, by clarifying the bridging role of interpersonal relationships between social media usage behavior and job satisfaction, this study offers a new perspective for theoretically exploring the deeper work outcomes brought about by social media behavior.

### 5.2. Practical Implications

#### 5.2.1. Organizational Level

Firstly, organizations should recognize that gender differences exist in social media use and its impact on job satisfaction. The study indicates that female employees are more adept at using social media to build and maintain interpersonal relationships, which in turn enhances their job satisfaction. Organizations should support female employees in utilizing this advantage while implementing targeted training programs to enhance male employees’ social media competencies, helping them better utilize social media to strengthen social interactions at work. Secondly, organizations should encourage employees to use social media for work-related communication and collaboration, while ensuring data privacy and security. By sharing work experiences and knowledge, social media can foster team collaboration, improve work efficiency, and enhance employees’ sense of belonging, thereby increasing job satisfaction. Lastly, organizations should foster a positive and supportive social media culture, encouraging employees to share positive experiences from both work and life. Interactive activities, such as likes and comments, can strengthen connections between employees, contributing to a better work atmosphere. At the same time, organizations should help employees use social media effectively, avoiding risks of information overload and work–life imbalance.

#### 5.2.2. Individual Level

Firstly, individuals should actively participate in discussions and sharing on social media, showcasing professional insights, which can help enhance personal image, expand social networks, and ultimately improve job satisfaction. Research shows that positive social media interactions can help individuals gain more social support, strengthening their career development. Secondly, individuals should improve their communication skills on social media, including effectively expressing opinions and appropriately using interactive tools. This will promote higher-quality social interactions, helping individuals build closer relationships with colleagues and gain more support at work. Lastly, individuals should use social media wisely, being mindful of its double-edged nature. While social media can enhance interpersonal relationships, it may also lead to information overload and work–life imbalance. Individuals should set reasonable limits on their usage time to ensure a balance between online interactions and real life, thus improving their quality of life and job satisfaction.

### 5.3. Research Limitations and Future Directions

While this study offers methodological and contextual innovations, several limitations warrant attention in future research. First, although the measurement methods employed in this study are supported by the existing literature, future research could consider adopting more comprehensive scales and supplementary measurement approaches to enhance the robustness of the findings. For example, more established multidimensional job satisfaction scales could be employed, along with objective data from social media platforms, to validate the measurement validity of sharing behavior. Second, while this study partially validates Demircioglu’s (2018) conclusion about public social media’s indirect influence on job satisfaction, it also finds that Moments sharing frequency can directly affect job satisfaction in specific contexts, which differs somewhat from previous findings. The influence of social media on job satisfaction appears to be contingent upon both usage contexts and platform-specific features, warranting further exploration and validation in future research. Furthermore, the findings regarding gender differences require deeper theoretical explanation. Future research could employ qualitative methods, such as in-depth interviews and case analyses, to explore how employees of different genders perceive and interpret social media’s influence on their job satisfaction, and how these differences are shaped by social gender roles. Additionally, future research should systematically investigate the potential adverse effects of social media use. Future research should consider longitudinal study designs to examine negative effects such as information overload and work burnout and explore the role of individual regulatory capabilities in mitigating these negative impacts. Additionally, attention should be paid to the “double-edged sword” effect of social media use, identifying the critical conditions where its impact shifts from positive to negative, thereby providing more detailed theoretical foundations for comprehensively understanding the mechanisms of social media’s role in the workplace.

## Figures and Tables

**Table 1:** Variable measurement descriptions.

Variable Type	Variable Name	Survey ID	Variable Description
Dependent Variable	Job Satisfaction	qg406	1 = Very dissatisfied; 2 = Dissatisfied; 3 = Neutral; 4 = Satisfied; 5 = Very satisfied
Core Independent Variable	Gender	gender	0 = Female; 1 = Male
Mediating Variables	Frequency of Moments Sharing	qu111	1 = Almost every day; 2 = 3–4 times a week; 3 = 1–2 times a week; 4 = 2–3 times a month; 5 = once a month; 6 = Once every few months; 7 = Never (reverse-coded)
	Quality of Social Relationships	qm2011	0 = Lowest score; 10 = Highest score
Control Variable	Age	age	Actual Age
	Education Level	edu_last	0 = Illiterate/Semi-illiterate; 3 = Primary School; 4 = Middle School; 5 = High School/Vocational School/Technical School; 6 = College; 7 = Undergraduate; 8 = Master; 9 = PhD; 10 = Never attended school (no samples in the analysis never attended school)
	Marital Status	qea0	1 = Single; 2 = Married; 3 = Cohabiting; 4 = Divorced; 5 = Widowed
	Health Status	qp201	1 = Very Healthy; 2 = Healthy; 3 = Relatively Healthy; 4 = Average; 5 = Unhealthy
	Local Income Status	qn8011	1 = Very Low, 5 = Very High
	Current Household Registration Status	qa301	1 = Agricultural Household; 3 = Non-agricultural Household; 5 = No Household Registration; 7 = Resident Household
	Direct Subordinates	qg17	1 = Yes; 5 = No
	Social Status	qn8012	1 = Very Low, 5 = Very High
	Weekly Working Hours (hours)	qg6	[0.1, 168]
	One-way Commuting Time (minutes)	qg3011	[0, 240]
	Night Shift Frequency	qg601	1 = Never required; 2 = Less than once a month; 3 = Once a month; 4 = Several times a month; 5 = Once a week; 6 = Several times a week; 7 = Daily
	Weekend Work Frequency	Qg602	1 = Never required; 2 = Less than once a month; 3 = Once a month; 4 = Several times a month; 5 = Weekly required
	Flexible Commuting Time	qg604	1 = No fixed working hours, completely arranged by oneself according to work needs; 2 = Basic fixed working hours with some flexibility; 3 = Completely fixed working hours
	Annual Work Income (RMB/year)	qg12	[0, 10,000,000]
	Employer Type	qg2	1 = Government/Party Organizations/Public Organizations; 2 = Public Institutions; 3 = State-owned Enterprises; 4 = Private Enterprises/Individual Businesses; 5 = Foreign/Joint Ventures; 6 = Other Types of Enterprises; 7 = Personal/Family; 8 = Private Non-enterprise Units/Associations/Guilds/Foundations/Village Committees

**Table 2:** Descriptive statistics.

Variable Code	Variable	N	Mean	SD	Min	Max
qg406	Job Satisfaction	4651	3.726629	0.8168488	1	5
gender	Gender	4651	0.5679887	0.4953998	0	1
qu111	Frequency of Moments Sharing	4651	4.932188	1.743059	1	7
qm2011	Interpersonal Relationships	4651	7.004426	1.644244	0	10
age	Age	4651	36.41555	10.21797	16	78
qa301	Household Registration Status	4651	2.443343	2.145821	1	7
qea0	Marital Status	4651	1.890935	0.6304947	1	5
qg2	Type of Employer	4651	3.518945	1.168318	1	8
qg3011	One-Way Commute Time	4651	21.47149	20.66696	0	210
qg6	Weekly Working Hours	4651	51.70882	17.16303	0.1	168
qg601	Frequency of Night Shifts	4651	2.351275	2.04557	1	7
qg602	Frequency of Weekend Work	4651	3.897309	1.524166	1	5
qg604	Flexibility of Working Hours	4651	2.392351	0.7181708	1	3
qg12	Total Work Income	4651	52,571.93	46,097.41	0	700,000
qg17	Presence of Subordinates	4651	0.179316	0.383658	0	1
qn8011	Income	4651	2.833392	0.8605868	1	5
qn8012	Social Status	4651	2.843839	0.8927095	1	5
qp201	Health Status	4651	2.669441	0.9974152	1	5
edu	Education Level	4651	4.197415	1.356362	1	8

**Table 3:** Baseline regression results.

Variable	(1)	(2)	(3)
	qu111	qm2011	qg406
Gender	−0.7249 ***	0.0317	−0.0843 ***
	(−17.7137)	(0.6889)	(−3.1104)
qu111		0.0451 **	0.0252 ***
		(2.5866)	(3.8996)
qm2011			0.0356 ***
			(3.7743)
Control Variables	YES	YES	YES
Provincial Fixed Effects	YES	YES	YES
Constant	5.0339 ***	5.8908 ***	3.9352 ***
	(14.2123)	(15.7077)	(28.3522)
N	4651	4651	4651
r2	0.0626	0.0969	0.1285

t statistics in parentheses, *p* < 0.01 **, *p* < 0.001 ***.

**Table 4:** Chain-mediated causal path analysis results.

Estimand	Estimate	SE	Lower	Upper	*p*
Pure Imputation Estimator					
Direct Effect (gender→qg406)	−0.091	0.023	−0.133	−0.039	0.000
gender→qm2011→qg406	0.004	0.005	−0.006	0.013	0.524
gender→qu111⇝qg406	−0.017	0.007	−0.031	0.004	0.02
Average Total Effect (ATE)	−0.103	0.023	−0.144	−0.056	0.000
Imputation-Based Weighting Estimator					
Direct Effect (gender→qg406)	−0.091	0.025	−0.135	−0.040	0.002
gender→qm2011→qg406	0.003	0.005	−0.007	0.014	0.506
gender→qu111⇝qg406	−0.016	0.008	−0.034	0.001	0.044
Average Total Effect (ATE)	−0.103	0.023	−0.144	−0.056	0.000

## Data Availability

The data for this study originate from China Family Panel Studies (CFPS), available in both Chinese and English. CFPS provides comprehensive user guidelines and resources. Access to the CFPS data requires an application, as direct sharing or redistribution by individuals is not permitted. Detailed application information and video instructions are available upon account registration at http://www.isss.pku.edu.cn/cfps/download/login, accessed on 26 December 2023.
